# Tuning the Pharmacokinetic Performance of Quercetin by Cocrystallization

**DOI:** 10.1021/acs.cgd.3c00590

**Published:** 2023-06-29

**Authors:** Molly M. Haskins, Oisín N. Kavanagh, Rana Sanii, Sanaz Khorasani, Jia-Mei Chen, Zhi-Yuan Zhang, Xia-Lin Dai, Bo-Ying Ren, Tong-Bu Lu, Michael J. Zaworotko

**Affiliations:** † Department of Chemical Sciences, Bernal Institute, University of Limerick, Limerick V94 T9PX, Ireland; ‡ School of Pharmacy, Newcastle University, Newcastle upon Tyne NE9 7RU, U.K.; § Tianjin University of Technology, Tianjin 300384, China; ∥ Sun Yat-Sen University, Guangdong 510275, China

## Abstract

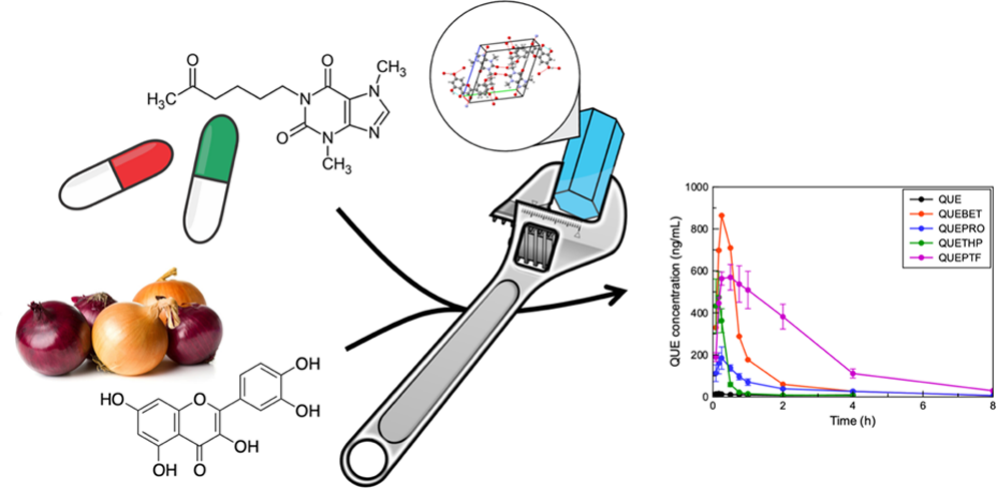

Quercetin (QUE) is a widely studied nutraceutical with a number of potential therapeutic properties. Although QUE is abundant in the plant kingdom, its poor solubility (≤20 μg/mL) and poor oral bioavailability have impeded its potential utility and clinical development. In this context, cocrystallization has emerged as a useful method for improving the physicochemical properties of biologically active molecules. We herein report a novel cocrystal of the nutraceutical quercetin (QUE) with the coformer pentoxifylline (PTF) and a solvate of a previously reported structure between QUE and betaine (BET). We also report the outcomes of in vitro and in vivo studies of QUE release and absorption from a panel of QUE cocrystals: betaine (BET), theophylline (THP), l-proline (PRO), and novel QUEPTF. All cocrystals were found to exhibit an improvement in the dissolution rate of QUE. Further, the QUE plasma levels in Sprague–Dawley rats showed a 64-, 27-, 10- and 7-fold increase in oral bioavailability for QUEBET·MeOH, QUEPTF, QUEPRO, and QUETHP, respectively, compared to QUE anhydrate. We rationalize our in vivo and in vitro findings as the result of dissolution–supersaturation–precipitation behavior.

## Abstract 2

Crystal engineering can combine western and traditional Chinese medicine to unlock the therapeutic potential of nutraceuticals.

## Introduction

In 2015, Professor Tu Youyou won the Nobel Prize for her discovery of the antimalarial drug artemisinin, traditionally extracted from the *Artemisia* annua plant.^1,2^ This discovery led to subsequent changes to the standard of care for malaria. This has renewed interest in nutraceuticals as a means to uncover a new generation of active pharmaceutical ingredients (APIs) for use in drug products. Nutraceuticals have been defined as a “food (or part of food) that provides medical or health benefits, including prevention and/or treatment of disease.”^3^ Despite the prevalence of nutraceuticals in many plant-based foods,^4^ poor bioavailability may prevent therapeutic in vivo concentrations from being attained, limiting their pharmacological response.

Quercetin (QUE), 3,3′,4′,5,7-pentahydroxyflavone, is a polyphenolic flavonoid nutraceutical found in various foods including citrus fruit, onions, and asparagus. It is widely recognized for its potential antioxidative properties^5^ and other potentially beneficial therapeutic effects such as anti-inflammatory, antiviral, anticarcinogenic, metal chelation, and cardioprotective effects.^6^ The extensive work on flavonoid analogues such as rutin (quercetin-3-*O*-rutinoside)^7,8^ attributes two reasons for this poor bioavailability, the rapid first-pass metabolism of QUE in the body and low aqueous solubility. One study reported that <1% of unchanged QUE was absorbed in the gastrointestinal (GI) tract,^9^ while other studies reported a significant decline in the pharmacological activity of the metabolites compared to its aglycon form (which confers increased water solubility).^10^ The Biopharmaceutical Classification System (BCS)^11^ is a useful guide for categorizing drug molecules based on their solubility and permeability properties. APIs classified in this way undergo different preformulation screening routes in order to improve their bioavailability. With an aqueous solubility of approximately ≤20 μg/mL, QUE is practically insoluble, equilibrating in water as quercetin dihydrate (QUE·2H_2_O).^12^ Despite its low bioavailability, QUE is known to possess a P_app_ of 10^–5^ cm/s.^13^ Consequently, QUE has been categorized as a BCS class II^14^ and IV^15^ molecule.

Various routine solid-form optimization strategies are employed to improve the solubility of APIs. These include salt formation (for ionizable compounds),^16^ solvate and polymorph screening,^17^ amorphous dispersions,^17^ and cocrystallization.^18^ Cocrystals are “solids that are crystalline single phase materials made up of two or more different molecular and/or ionic compounds generally in a stoichiometric ratio that are neither solvates nor simple salts^19^” and have proven to be a promising approach to increase the solubility and bioavailability of QUE.^9^ Some of us^9^ previously reported four new cocrystals of QUE, two containing caffeine (one anhydrous and one methanolate), which exhibited a significant increase in solubility (14- and 8-fold, respectively) and bioavailability (>10-fold) in rats compared to QUE dihydrate. Liu et al. reported a cocrystal of QUE with the highly soluble API, isoniazid.^20^ They observed that the dissolution rate of QUE increased by 52-fold and bioavailability by 29-fold. Other notable approaches taken to improve the solubility and bioavailability of QUE include microemulsion formulation,^21^ inclusion complexes,^22^ and solid dispersions.^23^ Despite the wealth of literature on QUE formulation approaches, there is a need to better understand the relationship between in vitro and in vivo behavior for cocrystal systems.

**Scheme 1:**
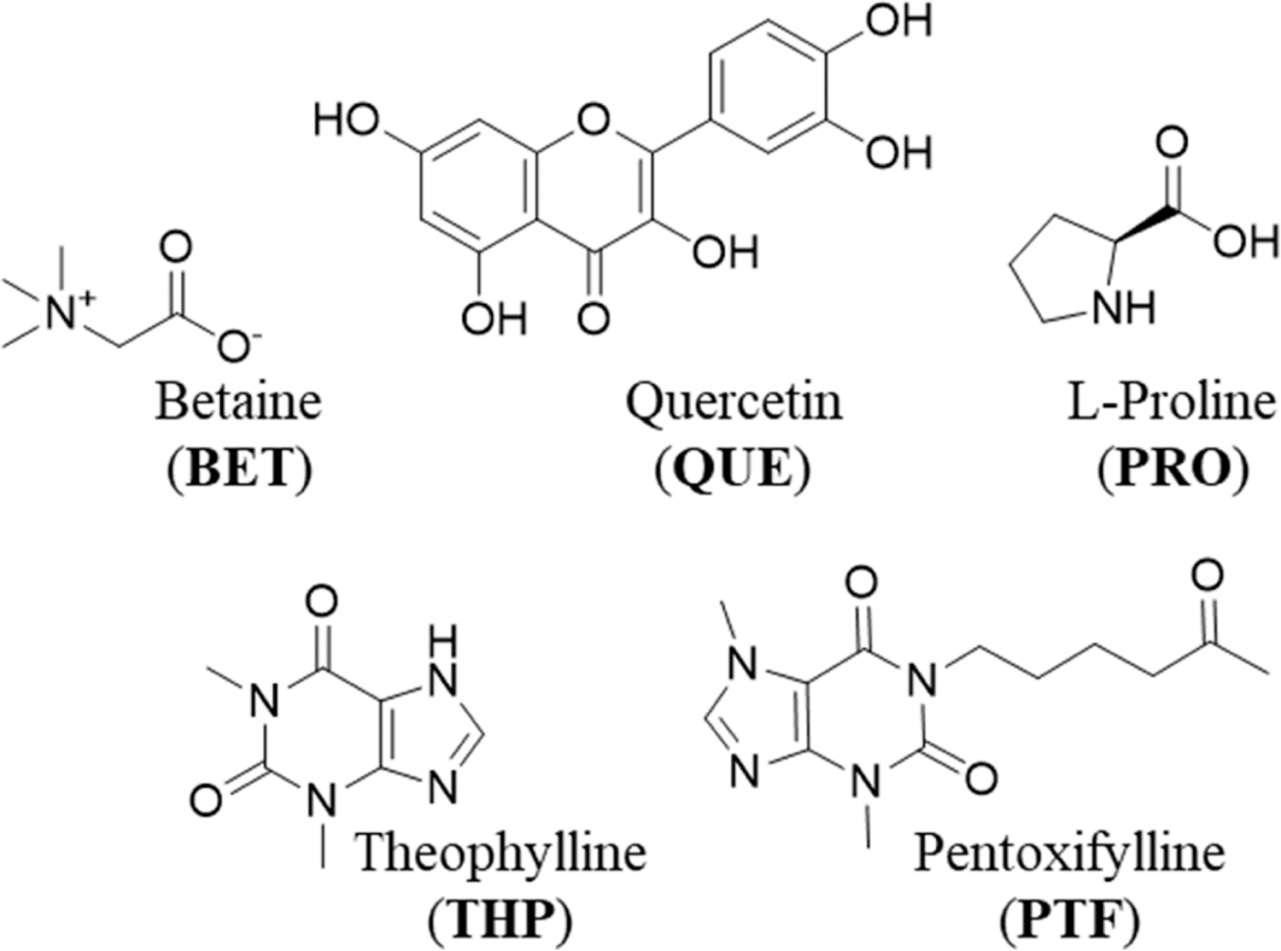
Structures and Abbreviations of the Molecular Coformers Used in This Study

Herein, we report the synthesis and pharmacokinetic characterization of cocrystal forms of QUE with four coformers, betaine (BET), proline (PRO), theophylline (THP), and pentoxifylline (PTF, Scheme 1). The physicochemical properties of all cocrystals were evaluated to assess their performance, as we hypothesized that cocrystallization could lead to improved bioavailability. In this work, we also explore the interface between in vitro dissolution kinetics and supersaturation for cocrystals of poorly soluble drugs like QUE, to understand the relationship between pharmacokinetic behavior and in vitro supersaturation, with the view that this may enable access to previously uncharacterized therapeutic activity.

## Materials and Methods

### Materials

QUE dihydrate was supplied by Bio-Technology Co., Ltd. and used for synthesis without further purification. Betaine (BET), theophylline (THP), pentoxifylline (PTF), l-proline (PRO), and all solvents and reagents were purchased from Sigma-Aldrich at the highest purity available and used as received. QUE anhydrous (US pharmacopeial standard) was purchased for dissolution and pharmacokinetic studies.

### Preparation of Cocrystals

#### Cambridge Structural Database (CSD) Analysis

The Cambridge Structural Database (CSD; version 5.43, November 2022 update) was used to search for archived quercetin cocrystal structures. The calculated powder X-ray diffraction (PXRD) patterns from the database were used to verify success in the synthesis of known cocrystal structures.

#### Quercetin/Pentoxifylline (QUEPTF)

##### Single Crystals

QUE·2H_2_O (34.0 mg, 0.101 mmol) and PTF (28.0 mg, 0.101 mmol) were dissolved in 3 mL of *iso*-propylalcohol (IPA) by mild heating at 40 °C. The solution was left to slowly evaporate at room temperature. Yellow needles of QUEPTF were harvested after 3 days. m.p.: 180 °C. These single crystals enabled the structure of QUEPTF to be determined by SCXRD.

##### Scale Up

QUE·2H_2_O (100.0 mg, 0.296 mmol) and PTF (82.4 mg, 0.296 mmol) were slurried in 6 mL of IPA for 24 h. The cocrystal product was filtered and air-dried. The bulk purity of the product was verified by PXRD.

#### Quercetin/Theophylline (QUETHP)

##### Solvent Drop Grinding (SDG)

QUE·2H_2_O (34.0 mg, 0.101 mmol) and THP (18.0 mg, 0.100 mmol) were added to a pestle and mortar with 500 μL of IPA and ground for 10 min, forming a homogenous powder. The yellow powder was analyzed by PXRD, TGA, and DSC. m.p.: 225 °C. The experimental PXRD data matched that calculated from the reported cocrystal (REFCODE: JATPIH).

##### Scale Up

QUE·2H_2_O (100.0 mg, 0.296 mmol) and THP (53.3 mg, 0.296 mmol) were slurried in 6 mL of IPA for 24 h. The cocrystal product was filtered and air-dried. The bulk purity of the product was tested by PXRD.

#### Quercetin/Betaine Methanolate (QUEBET·MeOH)

##### Scale Up

QUE·MeOH (100.0 mg, 0.296 mmol) and BET (35.0 mg, 0.299 mmol) were slurried in 6 mL of 1:1 MeOH/water for 24 h. The cocrystal product was filtered and air-dried. The bulk purity of the product was tested by PXRD, TGA, and DSC. Single crystals of sufficient quality could not be obtained to elucidate the structure. m.p.: 125 °C.

#### Quercetin/Proline (QUEPRO)

##### Scale Up

QUE·2H_2_O (100.0 mg, 0.296 mmol) and PRO (68.0 mg, 0.590 mmol) were slurried in 5 mL of ethanol/water for 24 h. The cocrystal product was filtered and air-dried. The bulk purity of the product was tested by PXRD and matched the calculated PXRD of the reported cocrystal (REFCODE/EJERES). m.p. 220 °C.

## Methods

### Powder X-ray Diffraction (PXRD)

PXRD studies of microcrystalline samples were performed in Bragg–Brentano geometry on a PANalytical Empyrean diffractometer (40 kV, 40 mA, Cu *K*_α1,2_ (λ = 1.5418 Å)). A scan speed of 0.5 s/step (6°/min) with a step size of 0.05° in 2θ was used at room temperature.

### Single-Crystal X-ray Diffraction (SCXRD)

Single crystals were manually selected and mounted with Paratone oil on a polymeric fiber. Data were collected on a Bruker Quest D8 diffractometer equipped with a Cu-sealed tube (Cu Kα radiation λ = 1.5418 Å), a Photon II CPAD detector, and an Oxford Cryosystem 800. Data were integrated with the APEX program suite and empirically corrected for absorption correction. The structure solution was conducted through direct methods in OLEX2. All heavy atoms were found on the electron density map and refined anisotropically against all *F*_obb_^2^. Hydrogen atoms were constrained through the riding model in their position as determined by an analysis of the distances between heavy atoms.

### Differential Scanning Calorimetry (DSC)

DSC analyses were carried out on a TA Instrument DSC Q20 under a sample purge of 50 mL min^–1^ of N_2_ with a heating rate of 10 °C min^–1^ for all cocrystals.

### Thermogravimetric Analysis (TGA)

TGA curves were performed on a TA Instrument Q50 TG under the flow of N_2_ with a heating rate of 10 °C min^–1^. The balance purge was 40 mL min^–1^ and the sample purge was 60 mL min^–1^ of N_2_.

### Accelerated Stability Testing

Accelerated stability testing was conducted by taking 100 mg of QUEPTF, QUETHP, QUEBET, and QUEPRO and placing them in a humidity chamber at 75% relative humidity achieved using a NaCl-saturated solution at 40 °C for 14 days. The samples were analyzed before and after testing by PXRD and TGA.

### Powder Dissolution Studies

Powder dissolution studies were carried out on QUE anhydrous, QUEBET·MeOH, QUEPRO, QUETHP, and QUEPTF. 20 mg equivalent of QUE anhydrous with a particle size between 75 and 100 μm (achieved by using a standard-mesh sieve) was added to 200 mL of phosphate buffer solution (PBS) 6.8 at 37 ± 0.5 °C with stirring at 100 rpm. 1 mL of aliquots were collected at the following time points: 1 3, 5, 10, 15, 30, 60, 120, and 240 min. Each aliquot was filtered through a 0.45 μm Whatman syringe filter. 500 μL of the filtrate was diluted with 500 μL of methanol and injected into an HPLC instrument. All dissolution experiments for QUE anhydrous and cocrystals were carried out in triplicate. The aliquots were analyzed using a Shimadzu (LC-20A) HPLC instrument with a Gemini C18 (250 × 4.6 × 5 μm^3^) column. The wavelength was set to 370 nm with an injection volume of 20 μL and a flow rate of 1 mL/min with an oven at 40 °C. The isocratic mobile phase comprised 0.1% orthophosphoric acid/methanol (65:35 v/v). QUE concentrations in the range of 25–250 μg/mL showed a good linear relationship (*R*^2^ > 0.99).

### In Vivo Pharmacokinetic Study

An in vivo pharmacokinetic study was performed on QUE anhydrous, QUEBET·MeOH, QUEPRO, QUETHP, and QUEPTF. Sprague–Dawley rats weighing 300 ± 20 g were purchased from Xinbo Pharmaceutical Research Co., Ltd. (Shandong, China) and housed in a temperature- and humidity-controlled room with a 12 h light/dark cycle and free access to food and water. Rats were fasted with free access to water for 18 h before drug administration. A 0.5% sodium carboxymethyl cellulose (CMC-Na) solution was selected as the gavage vehicle. The QUE formulations were delivered via oral gavage at a dosage of 100 mg of QUE/kg body weight. Approximately 200 μL of blood was collected at the following time points: 0, 5, 10, 15, 30, 45, 60, 120, 240, and 480 min. Blood samples were centrifuged (12,000 rpm, 2 min) immediately and a preservative solution at 10% (v/v) concentration was added to each separated plasma to ensure the integrity of QUE during storage. This preservative was composed of 20% ascorbic acid and 0.1% EDTA. The samples were stored at −80 °C until they were analyzed for their QUE content.

### Quantification of QUE in Rat Plasma

Xinbo Pharmaceutical Research Co., Ltd. (Shandong, China) analyzed the plasma samples for their QUE content using LC with tandem mass spectrometry. The standards were prepared as follows: A 1.00 mg/mL stock solution of QUE was accurately prepared in MeCN. Standards were prepared using the appropriate blank rat plasma with ascorbic acid and EDTA preservative. 50 μL of plasma was used as the aliquot volume and then 10 μL of standard/blank was added for all samples. Except for the double blanks, 200 μL of the internal standard (IS) spiking solution (50 ng/mL flurbiprofen in MeCN) was added to all samples. Tubes were then capped, vortexed for 2 min, and centrifuged at 12,000 rpm for 5 min. Approximately 200 μL of the supernatant was then transferred for analysis. The concentration range of the standard curve was 5–500 ng/mL of QUE. The results indicated that the standard curve performance was within the acceptable range for bioanalytical method acceptance (*R*^2^ > 0.99).

Plasma analysis was conducted using a Thermo Scientific Ultimate 3000 liquid chromatograph and an Applied Biosystems Sciex Triple Quad 5500 mass spectrometer with an electrospray ionization (ESI) source. The chromatographic separation was performed on a Waters Symmetry Shield RP8 (particle size 5 μm, 100 mm × 2.1 mm) column and the mobile phase consisted of acetonitrile/water with 0.2% acetic acid (60/40, v/v). The flow rate was 0.5 mL/min and the injection volume was 1 μL. The mass spectroscopy was performed in a negative mode. The spray voltage was −4500 V, and the capillary temperature was 550 °C. Multiple reaction monitoring (MRM) of MS/MS was used for specific detection of QUE and the internal standard (IS) by measuring the characteristic ion transitions of *m*/*z* 301.1 (parent ion) to *m*/*z* 151.1 (product ion) for QUE and *m*/*z* 243.0 (parent ion) to *m*/*z* 199.0 (production) for the IS flurbiprofen.

### Pharmacokinetic Calculations

Drug plasma concentration–time data were fitted using DAS 2.0 software, and pharmacokinetic parameters such as maximum concentration (*C*_max)_ and area under the curve (AUC) were obtained (Figure 3 and Table 2). All of the values were presented as mean ± standard deviation for four rats. Statistical data analysis was evaluated by IBM SPSS Statistics 22 software. Each QUE cocrystal was compared to the QUE dihydrate control, and differences were considered significant when *p* values were less than 0.05.

## Results and Discussion

### Crystal Structure Description

#### QUEPTF

**Figure 1:**
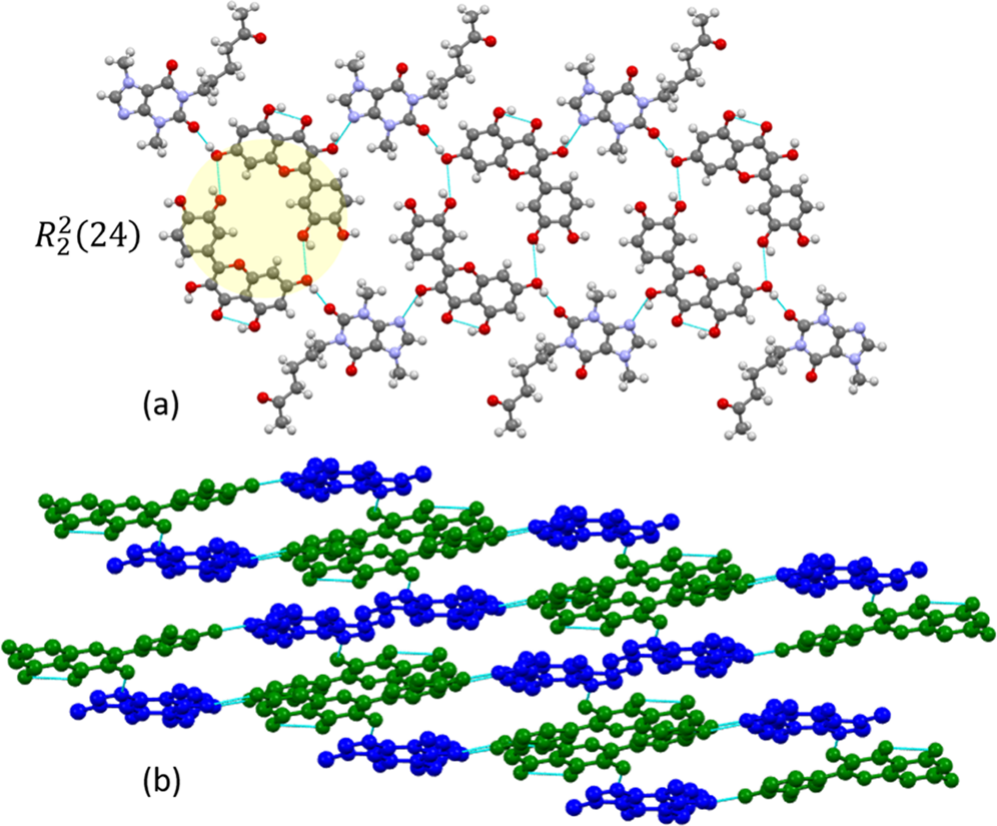
(a) Supramolecular sheet in QUEPTF along the *b*-axis and its graph set. The QUE and PTF molecules interact via O–H···N_arom_ and O–H···O H-bonds. (b) QUE is colored green and PTF is colored blue illustrating the H-bonding of PTF bridging the QUE dimers, viewed down the *c*-axis. Hydrogen atoms are omitted for clarity in (b).

The 1:1 cocrystal QUEPTF was crystallized in the triclinic space group *P*-1 with one molecule of QUE and one molecule of PTF in the asymmetric unit. One −OH of the catechol group and another −OH group of the chromone rings of QUE form a displaced dimer comprising two supramolecular homosynthons between the phenol groups of two QUE molecules, which can be described as an example of the *R*_2_^2^(24) graph set.^24^ This is highlighted in Figure 1a.

This same −OH group from the chromone rings forms a supramolecular heterosynthon by simultaneously H-bonding with the CO moiety of the amide ring of PTF (O–H···O=C, 2.687 Å). The second −OH of the catechol group forms a different heterosynthon with the ketone of PTF (O–H···O=C, 2.696 Å). The QUE dimers are slipped-stacked along the *b-*axis bridged by these PTF H-bonds on either side (Figure 1b). The N_arom_ of the imidazole ring of PTF hydrogen bonds to the ketone of QUE (O–H··· N_arom_, 2.787 Å). See Table S2 for the crystallographic table.

### Cocrystal Synthesis

Crystal forms were synthesized by the slurry; successful synthesis and cocrystal purity was confirmed by PXRD, TGA, and DSC respectively (Figure S1–4). PXRD diffractograms were compared to the calculated patterns for known phases and to the calculated pattern from SCXRD for QUEPTF.

### Thermal Analysis

TGA and DSC were used to analyze the thermal behavior of all four cocrystals. DSC thermograms illustrate the melting points of the four cocrystals, which are presented in Figures S5–S8. TGA profiles suggest the absence of solvent in QUEPTF and QUEPRO, and monohydrate and methanolate solvates of QUETHP and QUEBET, with desolvation (indicated at the onset temperature) occurring at 92 °C and 65 °C, respectively (Figure S5–S12); all forms decomposed beyond 250 °C. DSC confirmed that all solids employed in this study contained a single crystalline phase. QUEBET·MeOH appears to recrystallize immediately after the first melt and subsequently melt again at ca. 140 °C, which may indicate the recrystallization of an anhydrous phase. QUETHP melts at 160 °C with a recrystallization peak at 225 °C and a melt at 230 °C, indicating the melt of QUETHP hydrate and possible recrystallization subsequent melting of the anhydrous form.

Beyond an approximate numerical description, it is difficult to precisely determine what is degrading and when because of the broad degradation peaks that are incomplete even up to 400 °C. QUETHP appears to convert partially to QUE·2H_2_O during stability testing, which makes thermal analysis challenging for the post-accelerated stability testing sample. Pure QUETHP appears to lose some mass (3.768%, 0.189 mg) at <150 °C, which may correspond to adsorbed water. A second degradation peak occurs at 180 °C (10%, 0.5 mg), possibly indicating dehydration. Further, two degradation steps can be identified, but it is unclear what element of the cocrystal is degrading, as 30% remains at > 350 °C. The structurally similar cocrystal, QUEPTF, has two broad, overlapping degradation peaks with a first apparent weight loss of 40%, 2 mg from 250 °C, and a second from 325 °C.

QUEBET appears to lose some weight at temperatures around 100 °C (4.2%, 0.2 mg), suggesting desolvation, and appears to degrade further at 280 °C, which may correspond to the loss of betaine (20%, 1 mg). QUEPRO again has two degradation steps initially from 250 °C (30%, 1.5 mg) and another from 300 °C.

### Accelerated Stability Studies

QUEPTF, QUETHP, QUEBET·MeOH, and QUEPRO were stored in a 75% relative humidity chamber at 40 °C for 14 days. PXRD and TGA data were collected on samples before and after testing. After 14 days, the PXRD diffractograms indicated a possible phase change for QUETHP to QUE·2H_2_O, and no apparent phase change was observed for the remaining three samples tested (Figures S1–S4). TGA confirmed moisture uptake with QUETHP and no uptake with the remaining cocrystals (Figures S9–S12).

### Dissolution Experiments

**Figure 2:**
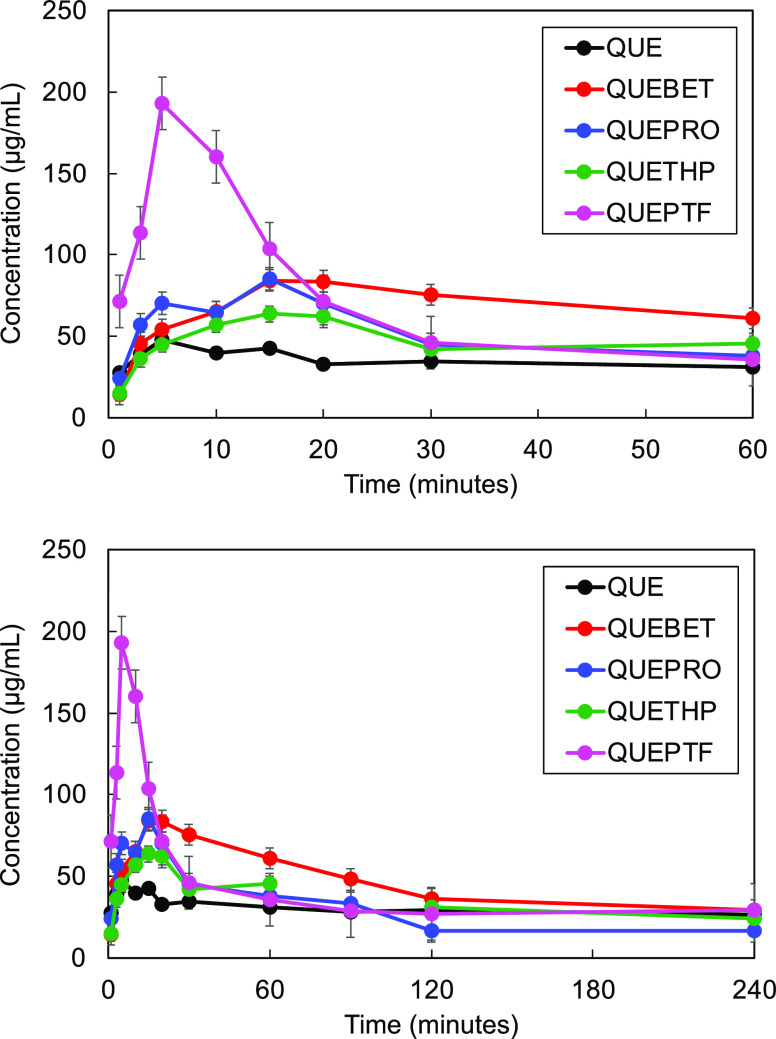
Dissolution profiles of QUE, QUEBET·MeOH, QUEPRO, QUETHP, and QUEPTF in PBS 6.8 over 1 and 4 h.

Some studies have reported that QUE falls below the limit of detection when performing dissolution tests in water or PBS buffer media.^10,25^ In this contribution, dissolution experiments were conducted on QUE anhydrous, QUEPTF, QUEBET·MeOH, QUEPRO, and QUETHP in triplicate. PBS pH 6.8 at 37 ± 0.5 °C was used as the dissolution medium, and no change in medium pH was observed during dissolution testing. Figure 2 represents the dissolution profiles of the four cocrystals and QUE dihydrate after 4 h.

All four cocrystals display a “spring” effect in their dissolution profiles.^26^ QUE anhydrous had a maximum solubility of 40.7 ± 1.7 μg/mL after 5 min before plateauing to 25.8 ± 1.6 μg/mL, corresponding to conversion to QUE·2H_2_O. All four cocrystals exhibited an increase in maximum solubility in comparison to QUE anhydrous within 15 min; QUETHP improved QUE solubility by 1.5-fold to 63.7 ± 3.9 μg/mL, while QUEPRO and QUEBET·MeOH increased by over 2-fold to 85.3 ± 1.7 and 85.5 ± 3.4 μg/mL, respectively. QUEPTF exhibited the highest maximum solubility of 192.9 ± 11.9 μg/mL, close to 5 times more than that of QUE dihydrate. Notably, QUEBET·MeOH exhibited a prolonged “parachute” effect. The residue at the end of the experiments was analyzed by PXRD to confirm that all four cocrystals had converted to QUE dihydrate as expected (Figure S1–4). These data suggest that all forms generated a supersaturated solution with respect to QUE·2H_2_O and that some cocrystal forms (e.g., QUEBET·MeOH and QUETHP) can sustain that supersaturation longer than others. For example, QUE from QUEPTF desaturated within 60 min, while QUE from QUEBET·MeOH appears to sustain supersaturation for up to 4 h.

Assuming that these in vitro dissolution plots correlate with the exposure of the GI tract to QUE, we present these data as AUC, *C*_max_, and *C*_min_ to gain insights into how these compounds might be expected to behave in vivo (Table 1). This reveals that in terms of total theoretical exposure, the cocrystal forms can be ranked as QUEBET·MeOH > QUEPTF > QUETHP > QUE > QUEPRO. In terms of *C*_max_, they can be ranked as QUEPTF > QUEPRO = QUEBET·MeOH > QUETHP > QUE.

**Table 1:** In Vitro Dissolution Data Viewed through a Pharmacokinetic Lens

parameter	QUE	QUEBET·MeOH	QUEPRO	QUETHP	QUEPTF
*C*_max_ (μg/mL)	47.8 ± 8.1	84.3 ± 8.26	85.3 ± 12.58	63.7 ± 0.78	192.87 ± 1.12
*C*_min_ (μg/mL)	25.85 ± 1.7	14.43 ± 7.74	16.53 ± 0.65	14.96 ± 1.86	26.99 ± 1.45
AUC_(0–240)_^a^ (mg·min/mL)	13.50	18.05	11.03	14.57	16.43

a AUC calculated by the sum of trapezoids from mean solubility data.

These results indicate that two distinct concentration profiles may be expected to arise in vivo, i.e., sustained supersaturation or a rapid, pronounced *C*_max_.

### In Vivo Pharmacokinetic Study of QUE

**Figure 3:**
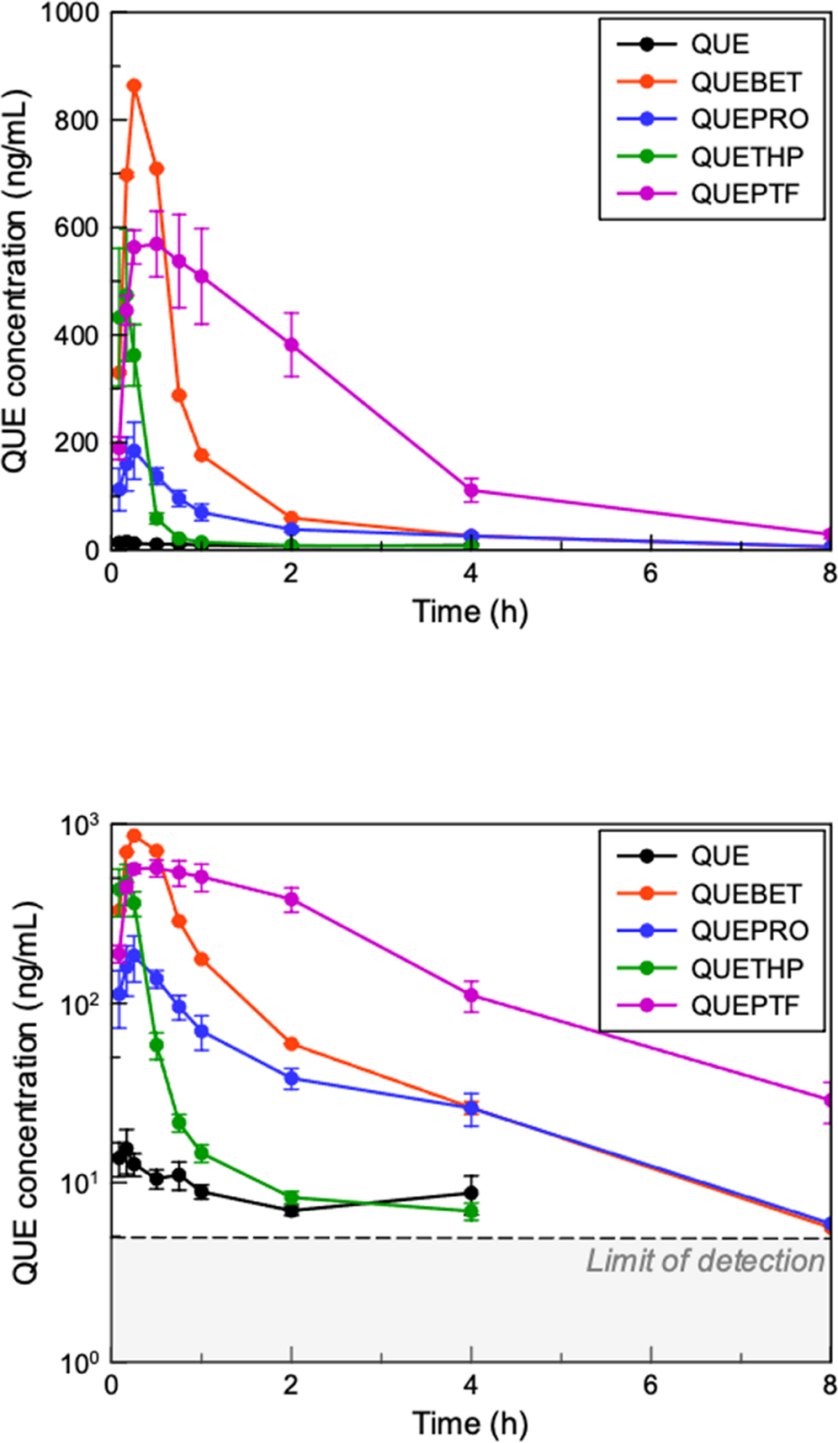
Mean QUE plasma concentration versus time for administration of QUE, QUEBET·MeOH, QUEPRO, QUETHP, and QUEPTF to Sprague–Dawley rats in linear and log scales.

The mean plasma concentration versus time profiles of QUE and the cocrystals when administered via a single oral gavage are illustrated in Figure 3 with detailed pharmacokinetic parameters listed in Table 2. QUE exhibited the lowest *C*_max_ of 18.7 ± 7.6 ng/mL after 15 min, while QUEBET·MeOH exhibited the highest *C*_max_ of 897.4 ± 231.6 ng/mL, a 48-fold increase in comparison to QUE. QUETHP exhibited a superior *C*_max_ value to QUE alone with a 27.6-fold increase. QUEPTF demonstrated a 33-fold increase in maximum solubility concentration compared to QUE2·H_2_O with 616.8 ± 129.5 ng/mL. QUEPRO had the lowest *C*_max_ out of all four cocrystals with 193.1 ± 113.9 ng/mL. Peak plasma concentrations for QUE and all cocrystals are reached after approximately 20 min, which rapidly declines within 8 h, corresponding to distribution and elimination processes. QUE and QUETHP reach the limit of detection beyond 4 h, and QUEPRO and QUEBET·MeOH reach this limit at 8 h.

**Table 2:** Pharmacokinetic Parameters for QUE and QUE Cocrystals^a^

parameter	QUE	QUEBET	QUEPRO	QUETHP	QUEPTF
*C*_max_ (ng/mL)	18.7 ± 7.6	897.4 ± 231.6^**^	193.1 ± 113.9	516.7 ± 252.5*	616.8 ± 129.5^**^
*T*_max_ (h)	0.4 ± 0.3	0.3 ± 0.1	0.4 ± 0.1	0.2 ± 0	0.5 ± 0.2
AUC(0–*t*) (ng*h/mL)	26.8 ± 11.5	743.0 ± 182.0*	262.8 ± 67.6*	184.4 ± 54.1*	1705.3 ± 224.0^**^

a (±S.D., *n* = 4) *p < 0.05 Cocrystals with Respect to QUE, **p < 0.01.

In terms of total exposure to QUE, QUEPTF enabled the highest absorption over the course of the experiment, with a 64-fold improvement in bioavailability versus the parent drug (AUC = 1705.3 ± 224.0 ng.h/mL). QUEBET·MeOH revealed the second highest AUC with 743.0 ± 182.0 ng.h/mL, close to a 28-fold increase in comparison to QUE. QUEPRO exhibited a 10-fold increase with 262.8 ± 67.6 ng.h/mL, whereas QUETHP exhibited a 7-fold increase compared to QUE with 184.4 ± 54.1 ng.h/mL. As predicted by the in vitro analysis, two distinct pharmacokinetic characteristics are conferred by the cocrystal forms. Supersaturation leads to a rapid increase in C_max_ or an overall increase in total exposure due to sustained supersaturation. The cocrystal forms can again be ranked in terms of *C*_max_ (QUEBET·MeOH > QUEPTF > QUETHP > QUEPRO > QUE) and AUC (QUEPTF > QUEBET·MeOH > QUEPRO > QUETHP > QUE).

QUE is a poorly soluble polyphenol nutraceutical with potential beneficial therapeutic effects ranging from anti-inflammatory and antioxidant to anticancer properties. Access to these properties is potentially limited by its poor solubility and bioavailability. Although QUE can be classified as either a BCS class II or a BCS class IV, BCS nomenclature may be inappropriate for nutraceuticals like QUE. The literature (and our findings) suggest that QUE does not meet the threshold of high permeability, as it has a bioavailability of ≪85% (i.e., BCS IV). However, one study finds a P_app_ of 10^–5^ cm/s for QUE (BCS II).^13^ While current guidance suggests that drug bioavailability can be used as a substitute for (and is sometimes preferred to) permeability due to the large inter-lab variation in traditional CaCo-2 models,^11,27^ the use of bioavailability in this context could have ruled out the rationale for improving QUE bioavailability by enhancing aqueous solubility. A further semantic point is that it is difficult to place nutraceutical solubility in the context of the BCS classes, as we do not know what the therapeutically effective dose is. This suggests the need for a modified developability framework for nutraceuticals or nomenclature consensus.^28^

Cocrystals have proven generally effective at modulating the physicochemical properties of APIs, including nutraceuticals.^29^ QUE is a particularly suitable candidate for crystal engineering due to its high propensity toward forming strong intermolecular interactions via H-bonds. Consequently, it is perhaps unsurprising that there are 40 QUE cocrystal structures deposited on the CSD, 11 are pharmaceutically relevant, as their coformers are either another APIs or a GRAS-listed coformer. The other 29 structures were prepared to develop crystal engineering principles through hierarchical and systematic studies (Table S1).

Interestingly, QUEPTF appeared to convert back into QUE dihydrate within 1h, while QUEBET·MeOH exhibited a “parachute” effect, transforming to QUE dihydrate at a slower rate. Zhang et al. reported the anhydrous form of QUEBET and conducted dissolution tests in PBS 6.8 where they also experienced a “spring effect” in their dissolution profile.^30^ They describe a “hover” phenomenon, where their cocrystal equilibrated at a higher concentration after 72 h, instead of conventionally returning to the QUE dihydrate concentration. They attribute this to the complexation of BET and QUE in solution, which may rationalize the slower transformation of the hydrated QUEBET·MeOH cocrystal to QUE2·H_2_O. Further, solubility studies have been performed for this cocrystal, which found evidence of complexation. However, in their study,^31^ they employ just 40 mL of PBS buffer with 100 mg equivalent of QUE. These equilibrium solubility measurements employ large amounts of the excess drug but do not detail the phase, which remains at completion. In contrast, our results do not suggest that the conditions enable complexation, as the QUE concentration after 240 min was equivalent to that of QUEBET, suggesting complete conversion of QUEBET to QUE·2H_2_O. Evidence of complexation would include the increased concentration of QUE compared to that of QUE·2H_2_O (converted from QUE anhydrate) for the QUEBET dissolution test. QUEPRO was evaluated previously in 0.5% Tween, finding that QUE possessed apparently superior dissolution behavior to QUEPRO.^32^ The contrast between those results and our own for this cocrystal system emphasizes the need for biorelevant conditions, as our methodology enabled us to identify that QUEPRO has advantages over QUE (i.e., it can generate a supersaturated solution with respect to QUE·2H_2_O), and these apparent in vitro advantages are also found to translate to the in vivo system. QUETHP and QUETHP.0.5H_2_O have been evaluated by other authors in a 0.5% Tween 80 medium.^33^ Although these authors found that QUETHP enhanced the dissolution rate and extent of solvation of QUE, they performed their experiments in media that contained surfactant and placed 500 mg of cocrystal into a 20 mL vessel. The conditions employed in these studies depart considerably from typical in vitro dissolution testing experiments, and our study represents conditions more closely reflecting the in vivo environment.

Of our new forms, QUEPTF can produce unpreceded in vivo exposure to quercetin in cocrystal forms to date. This pharmacokinetic behavior cannot readily be explained by the in vitro experiments. This raises the following question: why are levels raised well beyond the spring-and-parachute effect seen in the in vitro model? Indeed, in vitro results illustrate that QUE in solution released from the cocrystal forms returns to levels comparable to QUE2·H_2_O within around 30 min. There are two reasonable hypotheses to explain this pharmacokinetic profile in QUE cocrystals. (1) The cocrystals comprise substrates and inhibitors for a particular metabolic enzyme, which can competitively inhibit first-pass metabolism of each other during absorption and subsequent metabolic passes (e.g., quercetin inhibits CPY3A4, and xanthine drugs like THP, CAF, and PTF are all metabolized by CYP3A4^34,35^). (2) An intuitive possibility is that the shift in the circulation lifetime for these compounds is due to the presence of undissolved cocrystals that, upon eventual dissolution, enabled absorption; this is supported by our in vitro solubility data. This dissolution–supersaturation–precipitation behavior^36^ has also been observed previously for meloxicam cocrystals.^37^ However, both processes may be occurring concurrently.

Relative bioavailability (F_REL_) was used to evaluate the QUE cocrystals in this contribution to those reported in the literature. F_REL_ relates AUC_[cocrystal]_/AUC_[QUE]_. Some of this team previously published four novel cocrystals of QUE with isonicotinamide (INM), theobromine (QUETBR) as a dihydrate cocrystal, caffeine (QUECAF), and a methanolate solvate with caffeine (QUECAF.MeOH). Pharmacokinetic data are tabulated in Table 3. All three pharmacokinetic studies were conducted under the same conditions on the same breed of rats (male Sprague–Dawley). The only variant that may alter the pharmacokinetic profile of the cocrystal is the use of vegetable oil as the gavage vehicle in our group’s previous publication, while 0.5% CMC-Na was used in this study. Table 3 reveals that QUEPTF and QUEBET·MeOH have the largest *F*_REL_ compared to the other QUE cocrystals in the literature with F_REL_ of 63.6 and 27.7, respectively. The relative *C*_max_ and melting points of these cocrystals were also compared against those in the literature. These data indicate that QUEPTF and QUEBET·MeOH also exhibit the largest relative maximum concentration of QUE in the literature. This is stated precariously, however, as the QUE control in this study is lower than that in other studies.

**Table 3:** Table of Pharmacokinetic Parameters of the QUE Cocrystal Compiled from Data Presented in Table 2 and from the Literature for the Ease of Visualization

				
cocrystals	AUC (ng·min/mL^–1^)	*C*_max_ ng/mL	*F*_REL_	relative *C*_max_
QUE^9^	7.5	285		
QUECAF^9^	19.2	656	2.6	2.3
QUECAF.MeOH^9^	30.1	2612	4.0	9.2
QUEINM^9^	40.9	1401	5.5	4.9
QUETBR.H_2_O^9^	74.4	840	10.0	3.0
QUE^38^	467.3	911.7		
QUENIC^38^	728.4	5997.2	1.6	6.6
QUEPIC^38^	817.6	7832.0	1.8	8.6
QUE^25^	3880	540		
QUENIC^25^	15,200	710	3.91	1.31
QUE^39^	0.66	580		
QUEPZA^39^	17.07	8730	25.86	15.05
QUE^a^	26.8	18.7		
QUEPTF^a^	1705	616.8	63.6	32.9
QUEPRO^a^	262.8	193.1	9.8	10.3
QUEBET·MeOH^a^	743	897.4	27.7	48
QUETHP^a^	184.4	516.7	6.9	27.6

a Data obtained in this study.

## Conclusions

QUE is a polyphenol nutraceutical that exhibits beneficial biological activity but its utility as an API is hindered by low solubility and first-pass metabolism. Herein, we report the synthesis and characterization of two novel QUE cocrystals, one methanolate with BET and one anhydrate with PTF. Dissolution and pharmacokinetic studies were conducted on these novel solid forms as well as QUETHP and QUEPRO, two cocrystals previously reported. All four cocrystals were found to improve the solubility and bioavailability of QUE with QUEPTF exhibiting the largest improvement in bioavailability out of all QUE cocrystals reported to date. Both QUEBET·MeOH and QUEPTF might be regarded as candidates for further development based on their in vivo and in vitro performance. This study further validates that cocrystals can modulate the physicochemical properties of biologically active molecules in order to achieve improved bioavailability.

## Supporting Information Available

Table of QUE cocrystal deposited; PXRD plot QUE dihydrate (black), calculated PXRD, Differential Scanning Calorimetry, DSC of QUETHP, Accelerated Stability Testing, Overlaid TGAs of QUETHP hydrate before and after stability test (PDF)
