# Supplementary material for: Tuning the Pharmacokinetic Performance of Quercetin by Cocrystallization
Source: Cryst Growth Des. 2023 Jun 29;23(8):6059–66. doi: 10.1021/acs.cgd.3c00590 (PMC10401641; doi:10.1021/acs.cgd.3c00590)
Supplement: Supplementary file 1 — cg3c00590_si_001.pdf [file cg3c00590_si_001.pdf]

Supporting information

Tuning pharmacokinetic performance of quercetin by cocrystallisation

Molly M. Haskins, † Oisín N. Kavanagh, ◊* Rana Sanii, † Sanaz Khorasani, † Jia-Mei Chen, § Zhi-Yuan Zhang, § Xia-Lin Dai, # Bo-Ying Ren, § Tong-Bu Lu, § Michael J. Zaworotko †*

†*Department of Chemical Sciences, Bernal Institute, University of Limerick, V94 T9PX, Ireland.*

◊*School of Pharmacy, Newcastle University, Newcastle upon Tyne, NE9 7RU, UK.*

§*Tianjin University of Technology, Tianjin, 300384, China.*

#*Sun Yat-Sen University, Guangdong, 510275, China.*

*Corresponding authors: xtal@ul.ie and oisin.kavanagh@newcastle.ac.uk

CSD Analysis

Table S1. Table of QUE cocrystal deposited on the CSD (v5.3, March update). The pharmaceutically relevant structures are coloured in blue.

	REF CODE	Coformer (CF)	CF:QUE		REF CODE	Coformer (CF)	CF:QUE
**1**	ANAYUM	Praziquantel	2:1	**21**	YUFFIQ	4,4'-azopyridine	1:1
2	NAFZAY	Picolinic acid	1:1	**22**	YUFFOW	Phenazine 1,4-dioxane	1:1
**3**	EJERAO	D-proline	2:1	**23**	YUFGAJ	Phenazine	1.5:1
**4**	EJERES	L-proline	2:1	**24**	YUFGEN	Phenazine methanolate	3.5:1
5	MUPPOD	Theobromine dihydrate	1:1	**25**	YUFGIR	Phenazine monohydrate	1.5:1
6	NUTPEZ	Caffeine methanolate	1:1	**26**	YUFGUD	4,4'-bipyridine 2,2'-bithiophene	1:1
7	NUTPID	Isonicotinamide	1:1	**27**	YUFHAK	4,4'-bipyridine tetrathiofulvalene	3:2
8	NAFYUR	Nicotinamide	1:1	**28**	YUFHEO	1,2-bis(4-pyridyl)ethane	1:1
**9**	RUWHUN	Isonicotinic acid monohydrate	1:1	**29**	YUFHIS	*Trans*-1,2-bis(4-pyridyl)ethylene pyrene	3:2
**10**	TOHZUO	Betaine	2:1	**30**	YUFHOY	*Trans*-1,2-bis(4-pyridyl)ethylene	1:1
11	VIHJII	Pyrazinamide monohydrate	1:1	**31**	YUFPIA	Tetramethylpyrazine	3:1
**12**	YUFCOT	4,4'-bipyridine 1,4-dioxane	1:1	**32**	YUFPOG	Tetramethylpyrazine	2:1
**13**	YUFCUZ	4,4'-bipyridine THF	1:1	**33**	YUFPUM	Tetramethylpyrazine	1:1
**14**	YUFDAG	4,4'-bipyridine DMF	1:1	**34**	YUFQAT	Tetramethylpyrazine THF	1:1
**15**	YUFDEK	*Trans*-1,2-bis(4-pyridyl)ethylene	2:1	**35**	YUFQEX	4,4'-bipyridine 1,4-dioxane	1:1
**16**	YUFDIO	4,4'-bipyridine	1:1	**36**	YUFQIB	4,4'-bipyridine THF	1:1
**17**	YUFDOU	1,2-bis(4-pyridyl)ethane THF	1:1	**37**	YUGQOH	4,4'-bipyridine	1.5:1
**18**	YUFDUA	1,2-bis(4-pyridyl)ethane	2:1	**38**	YUFQUN	4,4'-bipyridine monohydrate	3:1
**19**	YUFFAI	1,2-bis(4-pyridyl)ethane	1:1	**39**	COLHIV	DABCO	2:3
**20**	YUFFEM	4,4'-azopyridine THF	1:1	**40**	QOLLUA	2,9-dimethyl-1,10-phenanthroline acetonitrile solvate	2:1

Crystallographic tables

Table S1. Crystallographic table of QUEPTF

Identification code	QUEPTF
Empirical formula	$C_{28}H_{28}N_4O_{10}$
Formula weight	580.54
Temperature/K	173.0
Crystal system	triclinic
Space group	P-1
a/Å	7.5750(10)
b/Å	13.8261(16)
c/Å	13.8438(16)
α/°	66.552(8)
β/°	89.064(9)
γ/°	86.690(9)
Volume/Å^3	1327.9(3)
Z	2
ρ_{calc}g/cm^3	1.452
μ/mm^{-1}	0.944
F(000)	608.0
Crystal size/mm^3	0.33 × 0.055 × 0.03
Radiation	CuKα (λ = 1.54178)
2Θ range for data collection/°	6.96 to 119.976
Index ranges	-8 ≤ h ≤ 8, -15 ≤ k ≤ 15, -15 ≤ l ≤ 14
Reflections collected	10729
Independent reflections	3808 [R_{int} = 0.2031, R_{sigma} = 0.2495]
Data/restraints/parameters	3808/0/387
Goodness-of-fit on F^2	1.015
Final R indexes [I>=2σ (I)]	R_1 = 0.1133, wR_2 = 0.2799
Final R indexes [all data]	R_1 = 0.2668, wR_2 = 0.3805
Largest diff. peak/hole / e Å^{-3}	0.42/-0.52

Powder X-ray Diffraction Data

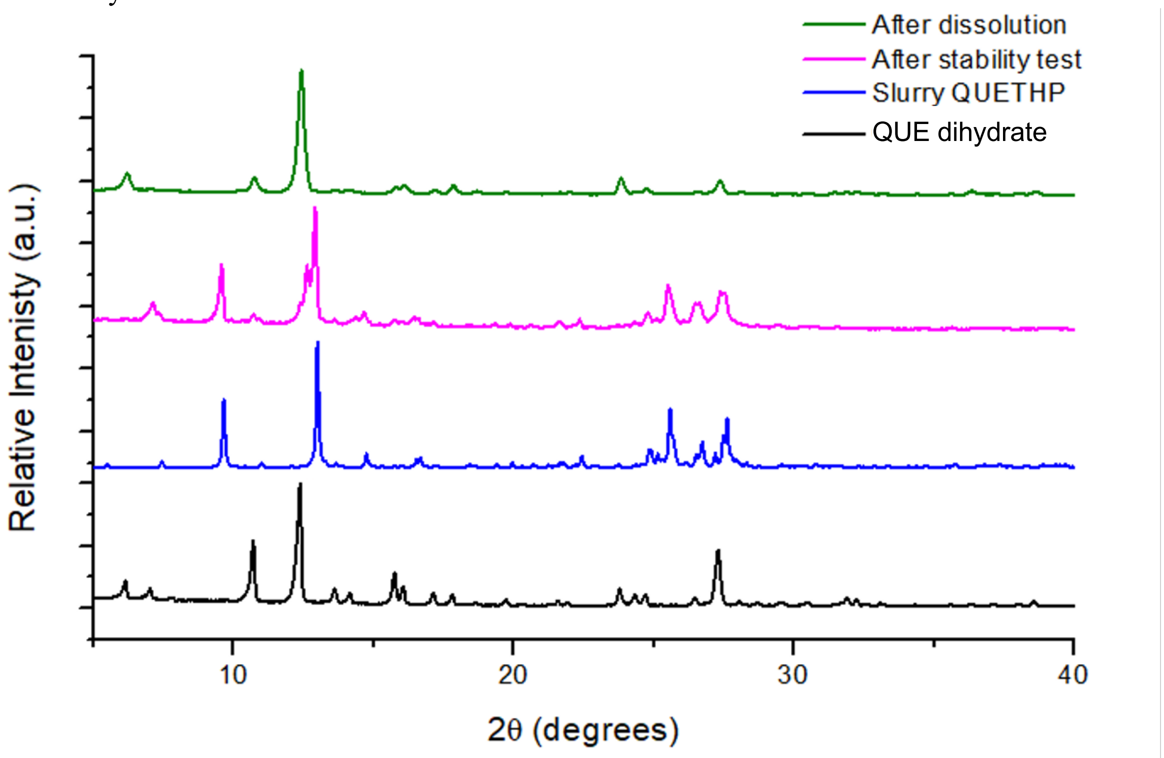

Figure S1. PXRD plot QUE dihydrate (black), calculated PXRD of QUETHP.H_2O (red), scale up of QUETHP.H_2O (blue), PXRD after accelerated stability (pink) and PXRD after dissolution studies in PBS 6.8 (green).

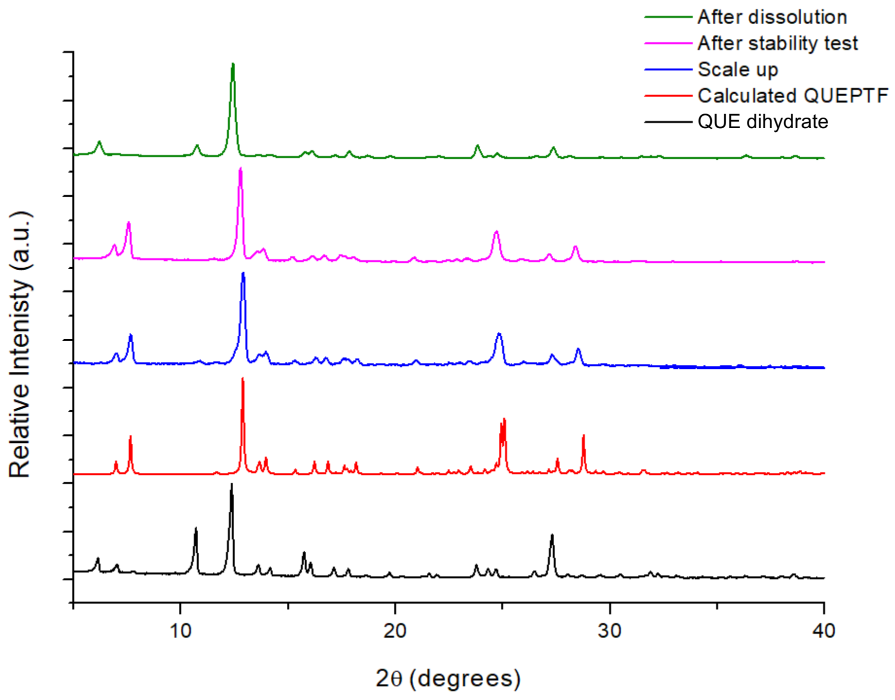

Figure S2. *PXRD plot QUE dihydrate (black), calculated PXRD of QUEPTF (red), scale up of QUEPTF (blue), PXRD after accelerated stability (pink) and PXRD after dissolution studies in PBS 6.8 (green).*

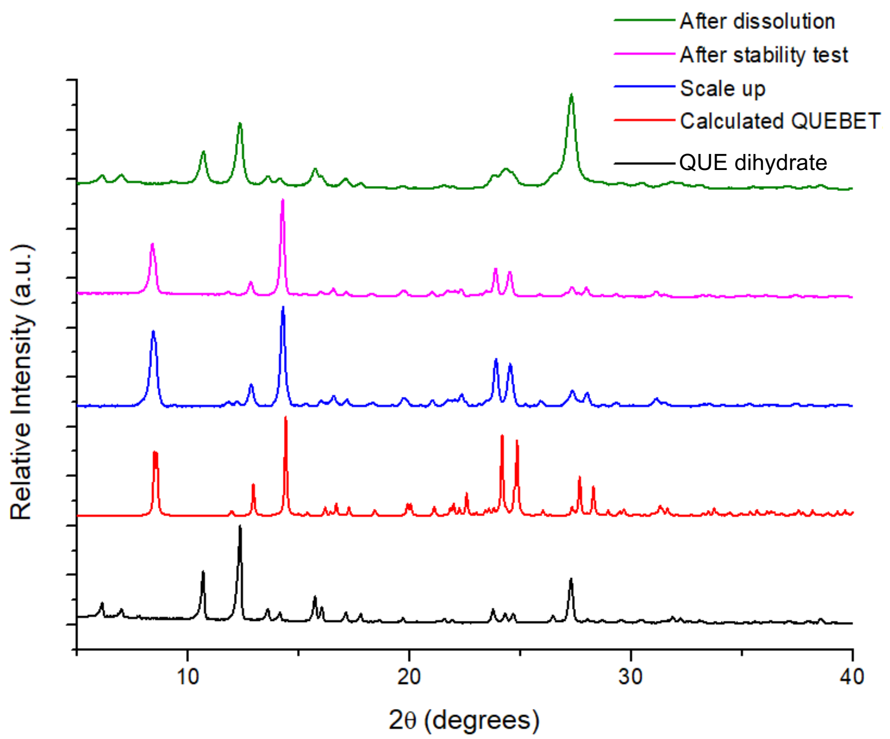

Figure S3. PXRD plot QUE dihydrate (black), calculated PXRD of QUEBET.H_2O (red), scale up of QUEBET.MeOH (blue), PXRD after accelerated stability (pink) and PXRD after dissolution studies in PBS 6.8 (green).

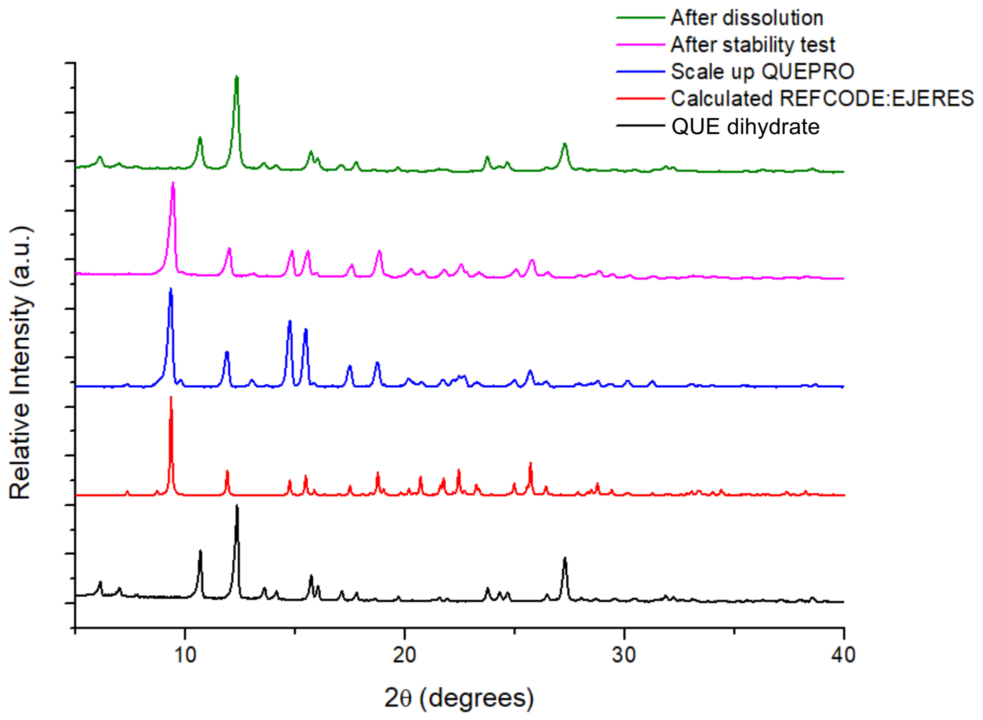

Figure S4. PXRD plot QUE dihydrate (black), calculated PXRD of QUEPRO (red), scale up of QUEPRO (blue), PXRD after accelerated stability (pink) and PXRD after dissolution studies in PBS 6.8 (green).

Differential Scanning Calorimetry

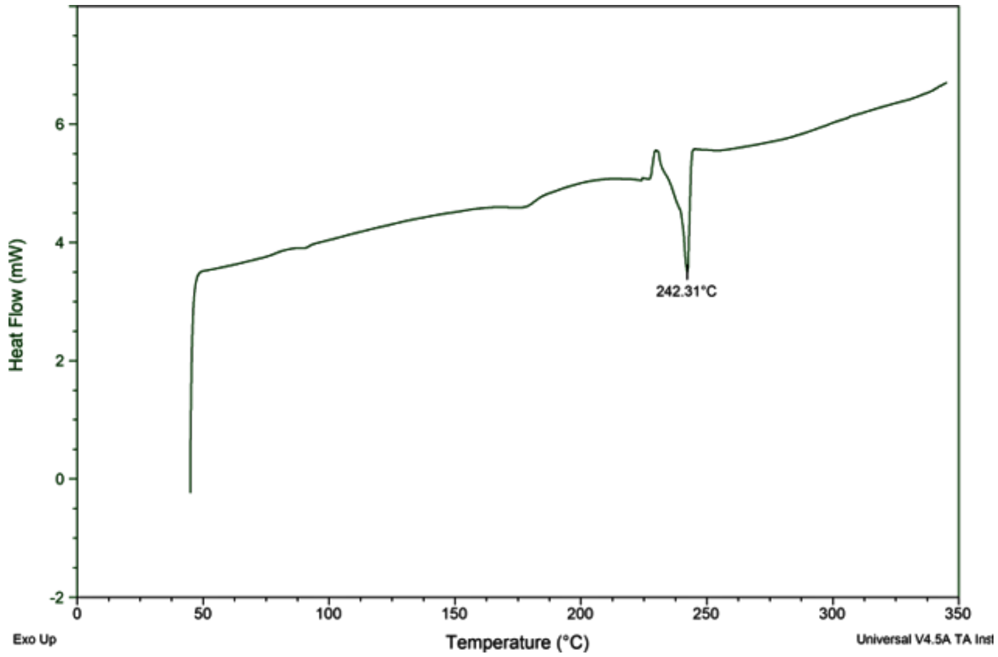

Figure S5. DSC of QUETHP, T_{onset} = 225 ºC.

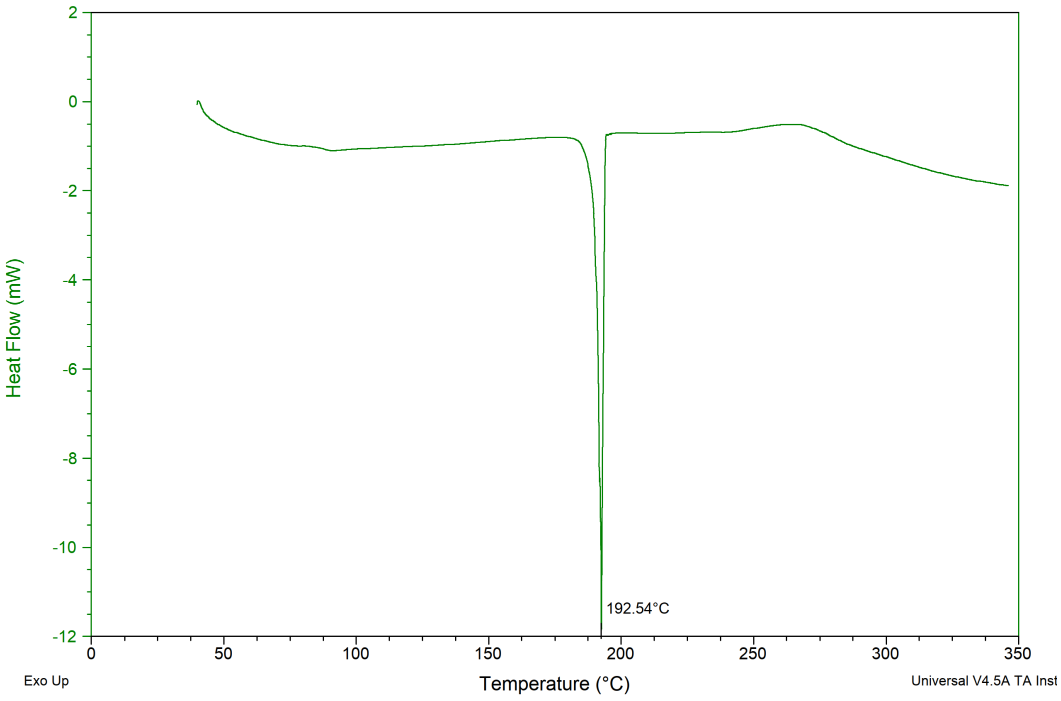

Figure S6. DSC of QUEPTF, T_{onset} = 180 ºC.

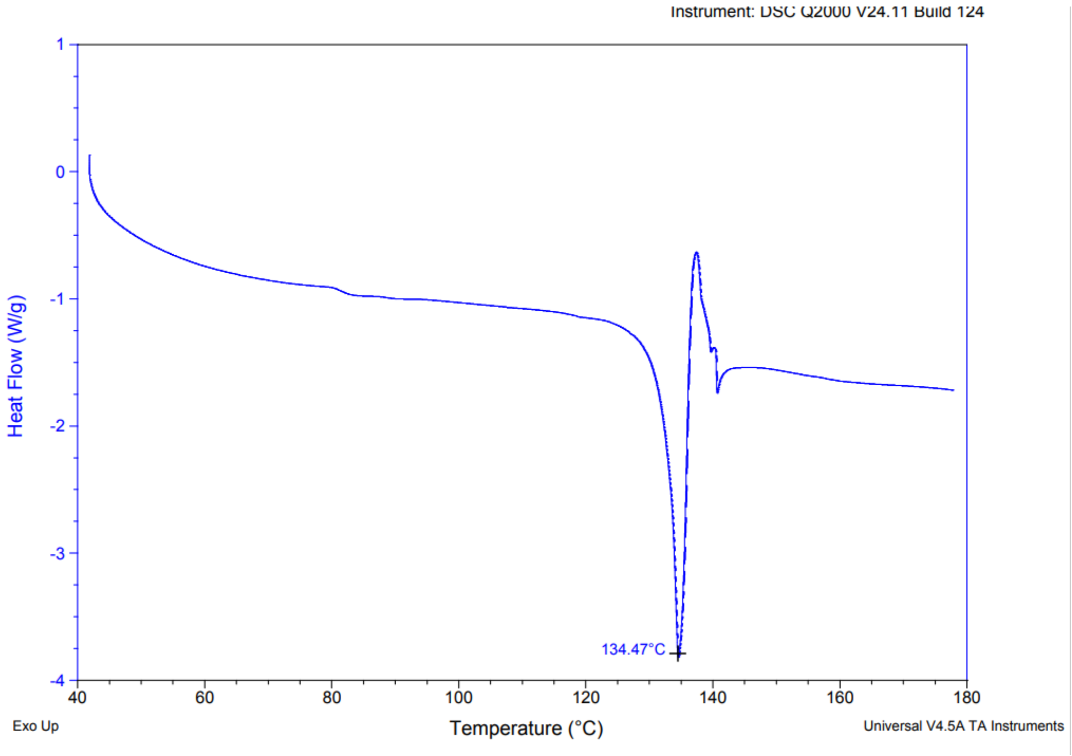

Figure S7. DSC of QUEBET.MeOH, T_{onset} = 125 ºC.

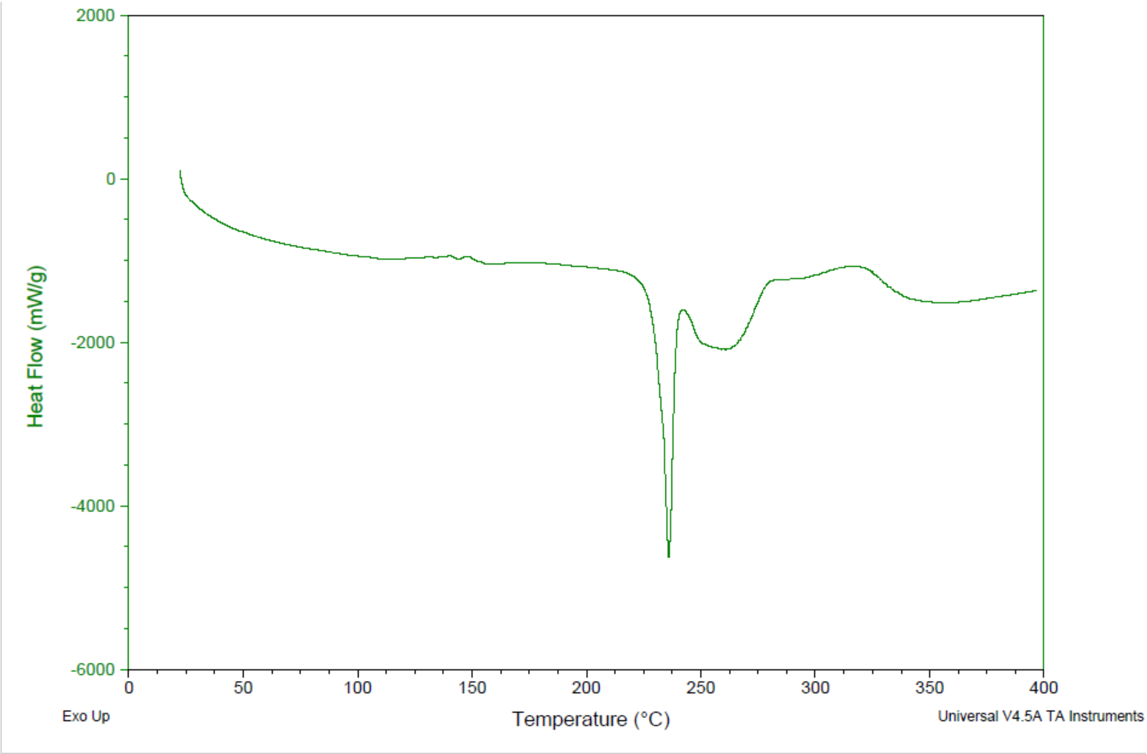

Figure S8. DSC of QUEPRO, T_{onset} = 220 ºC.

Accelerated Stability Testing (40°C/75% RH for 2 weeks)

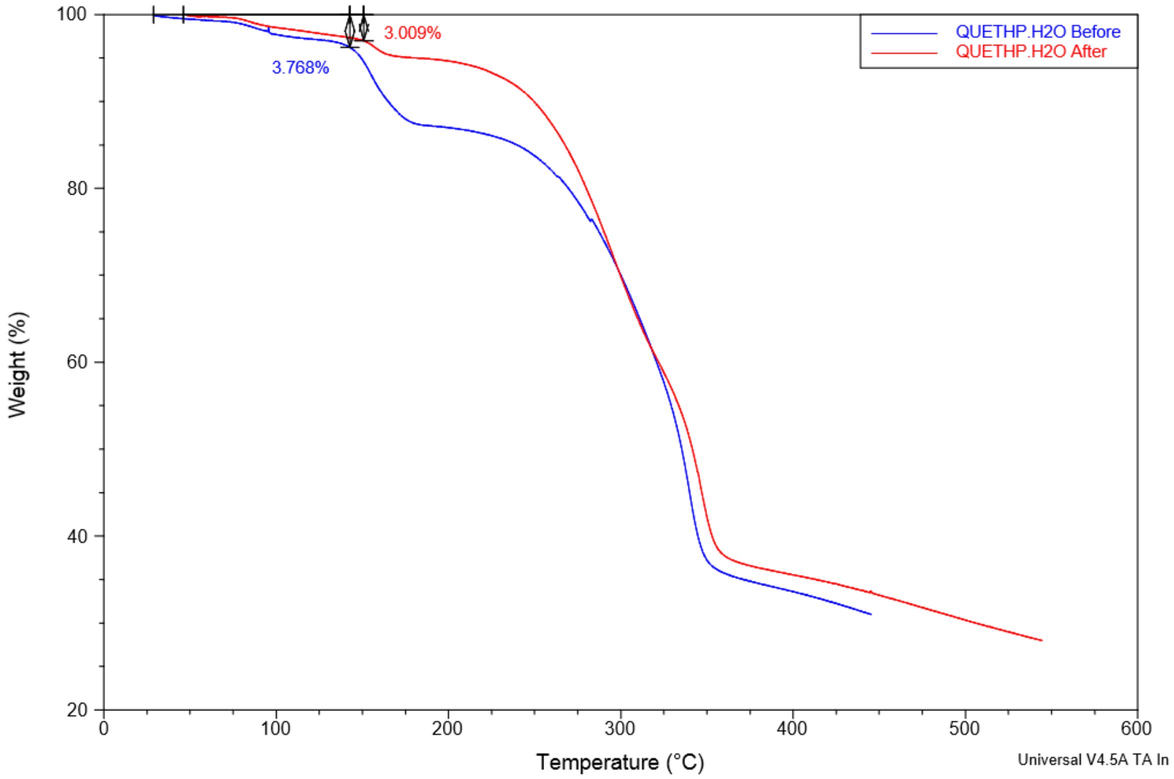

Figure S9. Overlaid TGAs of QUETHP hydrate before and after stability test

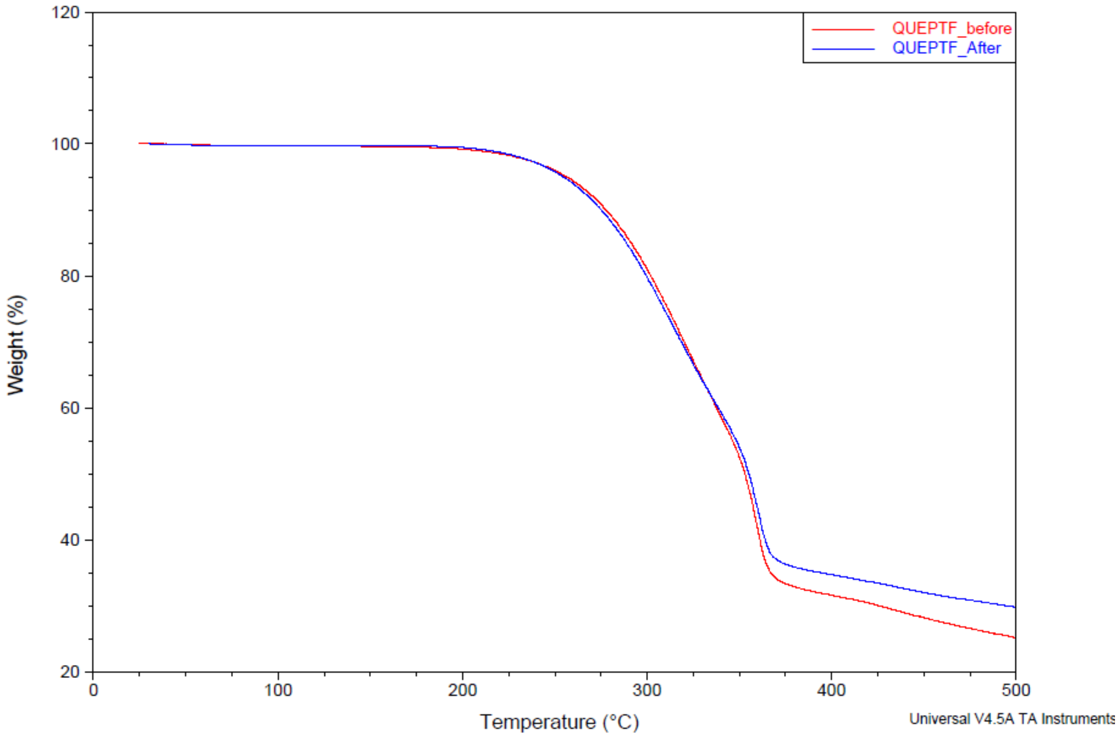

Figure S10. Overlaid TGAs of QUEPTF before and after stability test

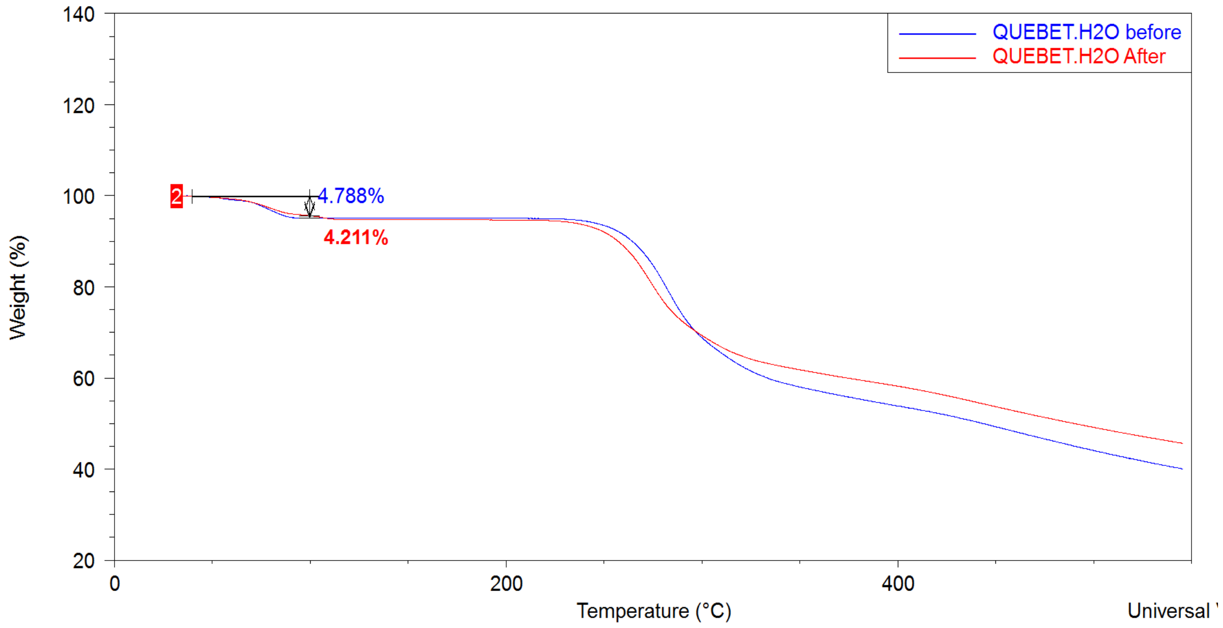

Figure S11. Overlaid TGAs of QUEBET.MeOH before and after stability test

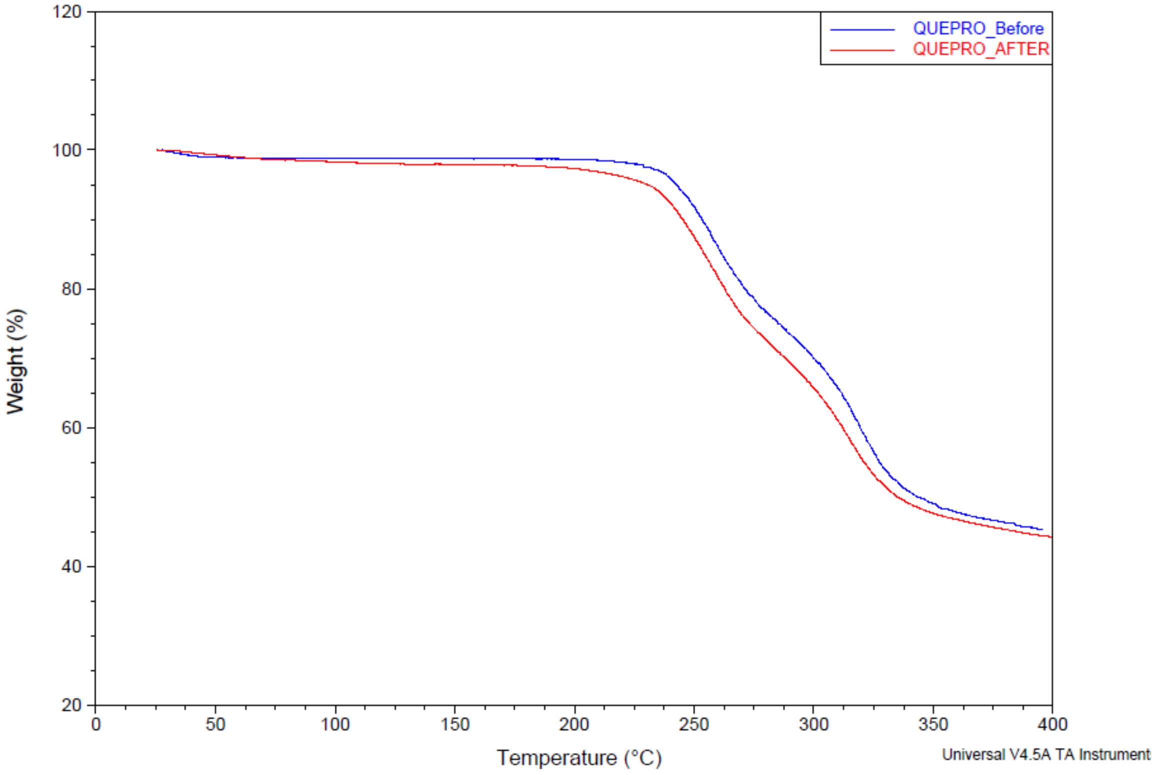

Figure S12. Overlaid TGAs of QUEPRO before and after stability test
